# Failure of a single day metronidazole desensitization protocol, and success of a modified two-day protocol in an outpatient setting

**DOI:** 10.1186/s13223-021-00640-4

**Published:** 2021-12-28

**Authors:** Julia A. Cahill, Preena Simritpreet Sahota, Manstein Kan

**Affiliations:** 1 Faculty of Medicine, University of Alberta, Edmonton, Canada; 2 Fraser Health Authority, Surrey, Canada; 3 Stellar Health, Surrey, British Columbia Canada; 4 Fraser Allergy, Langley, Canada

**Keywords:** Desensitization, Metronidazole allergy, Type 1 hypersensitivity, Outpatient allergy

## Abstract

**Background:**

True allergy to metronidazole, a common anti-infective in clinical practice, is rarely reported in the literature. In the case of *Trichomonas*, there are few alternatives to the nitrimidazole class of drugs, and the alternatives that do exist are associated with worse clinical outcomes. Accordingly, for the rare patients with Type 1 hypersensitivity reactions to metronidazole but compelling need, desensitization protocols have been adapted previously. Reactions during these protocols appear common. Patients in previous regimens have required higher level care for observation, which is costly and resource-intensive.

**Case presentation:**

We report here on a successful outpatient two-day regimen for metronidazole desensitization. Our patient had compelling indication for metronidazole, but reacted after receiving the very first dose of a previously described desensitization protocol. Accordingly, the protocol was adapted further. Despite this, she went on to develop objective hives prior to reaching the full intended dose. With appropriate symptom management and pre-medication on the second day in clinic, she was successfully desensitized and able to complete a week of full-dose metronidazole. No acute care resources were needed.

**Conclusion:**

We propose this two-day desensitization regimen for patients who react during the previously described desensitization protocols. This regimen was effective and safe, and did not necessitate the use of acute-care resources. Two-day desensitization protocols while relatively uncommon, can be successful.

## Background

There are few treatment options for *Trichomonas vaginalis*, beyond the nitroimidazole class of drugs. Generally, metronidazole and tinidazole are well-tolerated, with the exception of gastrointestinal side-effects [1]. Anaphylaxis and hypersensitivity have been reported in the literature in rare cases.

There are previously proposed protocols available for metronidazole desensitization. In 1991, Kurohara et al. described successful use of an oral regimen for desensitization to metronidazole [2]. In 1996, Pearlman et al. described effective intravenous desensitization to metronidazole [3]. Both of these protocols involved desensitization over the course of hours, with oral doses given in sequential doses 1 h apart, and intravenous doses given in sequential doses 15–20 min apart. The overall desensitization took less than one day for each protocol. In 2014, Grendelman et al. adapted the oral protocol initially described by Kurohara, to include more intermediate doses and a slower escalation to full oral dosing [4]. We describe here the case of a patient who reacted after the first dose of the Grendelman et al. protocol, and who reacted again later in the same day, necessitating a two-day desensitization protocol, which was successfully completed in a community outpatient clinic. Using this adapted regimen, the patient was able to complete her treatment course.

## Case presentation

A 50 year old female was referred to our outpatient clinic for evaluation of an allergic reaction to metronidazole. Within 20 min of receiving a therapeutic dose of metronidazole 500 mg to treat a *Trichomonas vaginalis* infection, she had experienced hives and throat swelling, which resolved with diphenhydramine. She had similarly reacted years prior, when she was prescribed metronidazole for an episode of diverticulitis. Past medical history was relatively unremarkable, though she did note diverticulitis, hypertension, fibromyalgia, chronic fatigue, and a previous hysterectomy. Concomitant chronic medications included amlodipine, perindopril, and boric acid. She had a history of allergic reaction to ranitidine, with documented reaction of throat tightness and hives. The patient was experiencing significant discomfort from her infection, and after discussion of the risks and benefits in our clinic, elected to undergo desensitization to metronidazole accordingly.

The modified oral desensitization protocol adapted by Grendelman et al. in 2014 was undertaken. We collaborated with a local community compounding pharmacy, and asked for their help in providing compounded suspensions of oral metronidazole to undertake the desensitization protocol. The compounding pharmacy provided a suspension of oral metronidazole 50 mg/mL, which was further diluted down prior to administration. To obtain the smallest doses early on in the protocol (Table 1), small volumes were diluted serially. Doses were administered in 15 min increments. After the first dose of 0.0025 mg, our patient reacted. Accordingly, Grendelman et al.’s protocol was further adapted for slower dose escalation (Table 1). Dilutions were calculated in writing prior to administration of each dose, to ensure mathematical accuracy. Our patient tolerated the escalating doses on day 1 until she reached a dose of 250 mg, at which point she developed objective hives without other systemic symptoms of anaphylaxis. She was given bilastine 20 mg and monitored until her hives resolved, and was provided a prescription for an adrenaline auto-injector to carry overnight.

**Table 1:** Metronidazole desensitization protocols

Dose	Kurohara et al. [2]	Grendelman et al. [4]	Current modified regimen
1	0.0025 mg	0.0025 mg	0.0025 mg
2	0.025 mg	0.025 mg	0.01 mg
3	0.25 mg	0.25 mg	0.02 mg
4	2.5 mg	2.5 mg	0.025 mg
5	25 mg	5 mg	0.25 mg
6	250 mg	10 mg	2.5 mg
7	500 mg	25 mg	5 mg
8	1000 mg	50 mg	10 mg
9		100 mg	25 mg
10		250 mg	50 mg
11		500 mg	100 mg
12		1000 mg	250 mg

			Day 2: premedication with oral anti-histamine
13			250 mg
14			500 mg

She returned the following day for further desensitization. Her hives went away the preceding evening. She reported some non-specific back pain. She had been provided with a tablet of bilastine 20 mg to take prior to returning on day 2. On day 2, she was given 250 mg and then 500 mg of metronidazole, from the original 50 mg/mL suspension obtained from the pharmacy, and monitored. She did not experience any reaction or discomfort. She was instructed to carry the adrenaline auto-injector with her for the week, and to complete a 7 day course of metronidazole 500 mg twice daily without interruption. She did this successfully, and her infection was eradicated.

## Discussion

Desensitization to substances for Type 1 hypersensitivity reactions is best described in the literature with penicillin-derivatives and aspirin; there are relatively few cases described for metronidazole [2–5]. Allergic reaction to nitroimidazoles is uncommon, but presents significant challenges for patients with *Trichomonas vaginalis*, as second-line treatments are associated with significantly higher failure rates [1]. While a recent study described desensitization to tinidazole [6], tinidazole is not available in Canada, limiting therapeutic options for Canadians.

As noted by Grendelman et al. in their protocol, there are few protocols available in the literature for metronidazole desensitization, and slower titrations are needed for patients who are higher risk, or those who react early on. Grendelman et al. had modified Kurohara’s protocol, as their patients had reacted to the lower initial doses in the protocol. Similarly to Grendelman’s study, our patient reacted early on, to the very first dose in the protocol by Kurohara et al. Even after modification of Grendelman’s protocol and slower uptitration in dosing, our patient developed objective hives at the dose of 250 mg, and necessitated antihistamine with monitoring. She could not complete the desensitization over the course of a single day, as is usually done in desensitization protocols, given concerns that patients may re-sensitize if there is sufficient time lapses between dose escalations. In our protocol, despite the time lapse between day 1 and resuming the protocol on day 2, our patient was able to resume at 250 mg on day 2 without evidence of re-sensitization and reaction.

In the few cases of metronidazole desensitization described in the literature, it seems common that patients react during desensitization, and this can have significant implications. In Grendelman’s protocol [4], their patient actually had to be desensitized in the ICU, as she had reacted in clinic with a pruritic rash. This is much more resource-intensive than desensitization in private clinics. With pre-emptive administration of oral antihistamine, despite her reaction, our patient was able to complete the desensitization the following day in our outpatient clinic, and successfully eradicate her infection with a 7 day course of oral metronidazole at full dosing. Thus, despite her reaction, a two-day regimen, which has not been previously described, successfully desensitized our patient without necessitating acute care resources. This may have important clinical implications for resource utilization, and during the COVID-19 pandemic, may help mitigate risk of spread by avoiding unnecessary hospital visits.

While desensitization in a hospital setting is the most ideal, it is not always practical given constraints in space and resources. Many patients welcome the convenience of desensitization in clinic over an acute care centre. When desensitization is undertaken in an outpatient setting, it is important that clinicians first evaluate the risk of doing so based on several factors, including but not limited to the severity of their initial reaction, patient comorbidities, clinician and patient comfort, and the clinic’s ability to safely manage a more serious reaction. In our case, our patient had been counselled on the possible risks as well as on our clinic management strategies. A shared decision-making approach was used to determine that the clinic setting would provide adequate support for her, as well as convenience.

## Conclusions

While metronidazole allergy in the literature is rarely described, for those with reported allergies, it does not seem uncommon to react during desensitization. For patients experiencing noticeable symptoms during metronidazole desensitization on day 1, a two day regimen with pre-medication adapted to patient’s tolerance may successfully be used to desensitize patients in an outpatient setting. Our patient went on to tolerate a full 7 day course at usual dosing, despite reacting twice during the conventional desensitization protocol. As our proposed two-day titration enabled achievement of a full therapeutic dose, it is worth trialling in patients who react during desensitization with previously described protocols.

## Data Availability

Not applicable.
